# Antibody humanization by molecular dynamics simulations—*in‐silico* guided selection of critical backmutations

**DOI:** 10.1002/jmr.2527

**Published:** 2016-01-08

**Authors:** Christian Margreitter, Patrick Mayrhofer, Renate Kunert, Chris Oostenbrink

**Affiliations:** ^1^ Institute of Molecular Modeling and SimulationUniversity of Natural Resources and Life SciencesMuthgasse 18Vienna1190Austria; ^2^ Department of BiotechnologyUniversity of Natural Resources and Life SciencesMuthgasse 18Vienna1190Austria

**Keywords:** molecular dynamics, antibody humanization, conformational clustering, binding affinity, GROMOS

## Abstract

Monoclonal antibodies represent the fastest growing class of biotherapeutic proteins. However, as they are often initially derived from rodent organisms, there is a severe risk of immunogenic reactions, hampering their applicability. The humanization of these antibodies remains a challenging task in the context of rational drug design. “Superhumanization” describes the direct transfer of the complementarity determining regions to a human germline framework, but this humanization approach often results in loss of binding affinity. In this study, we present a new approach for predicting promising backmutation sites using molecular dynamics simulations of the model antibody Ab2/3H6. The simulation method was developed in close conjunction with novel specificity experiments. Binding properties of mAb variants were evaluated directly from crude supernatants and confirmed using established binding affinity assays for purified antibodies. Our approach provides access to the dynamical features of the actual binding sites of an antibody, based solely on the antibody sequence. Thus we do not need structural data on the antibody–antigen complex and circumvent cumbersome methods to assess binding affinities. © 2016 The Authors Journal of Molecular Recognition Published by John Wiley & Sons Ltd.

## Introduction

Monoclonal antibodies (mAbs) represent the fastest growing class of biotherapeutic proteins, with US sales reaching $24.6 billion in 2012 (Aggarwal, 2014). As of January 2015, IMGT® (Lefranc *et al.*, 2005), the international ImMunoGeneTics information system (http://www.imgt.org) listed 36 mAbs approved by the FDA or EMEA for human therapeutic use (Poiron *et al.*, 2010). Only 12 of the approved antibodies are of human origin, whereas the majority represents rodent (3), chimeric (7) or humanized antibodies (14), all containing non‐human sequences. However, antibodies that are derived from non‐human organisms and are applied in human therapies may lead to the human anti‐mouse antibody response. Because of their foreign characteristics, they can lead to adverse and potentially harmful side‐effects because of altered efficacy and pharmacokinetics (Schroff *et al.*, 1985; Shawler *et al.*, 1985; Sgro, 1995). This indicates the importance for techniques to reduce immunogenicity of antibodies by making them more human‐like.

Traditional methods to reduce the risk of severe immunogenic responses to therapeutic antibodies are based on chimerization (Boulianne *et al.*, 1984; Morrison *et al.*, 1984; Neuberger *et al.*, 1985) or complementarity‐determining region (CDR)‐grafting (Jones *et al.*, 1986; Riechmann *et al.*, 1988; Queen *et al.*, 1989). Further advanced protocols include resurfacing (Roguska *et al.*, 1994), framework shuffling (Dall'Acqua *et al.*, 2005), human content optimization (Lazar *et al.*, 2007), superhumanization (Tan *et al.*, 2002; Hwang *et al.*, 2005), screening of human antibody libraries (Winter *et al.*, 1994; Low *et al.*, 1996; Bradbury *et al.*, 2011) or immunization of transgenic mice (Brüggemann *et al.*, 1991; Taylor *et al.*, 1992; Mendez *et al.*, 1997; Lonberg, 2005). The primary purpose of all of these methods is to keep the risk of adverse side‐effects (Hansel *et al.*, 2010) at an absolute minimum. Applied to human subjects, the engineered therapeutic antibody must not trigger any critical human anti‐mouse (Schroff *et al.*, 1985; Shawler *et al.*, 1985; Sgro, 1995) or human anti‐chimeric (Khazaeli *et al.*, 1994) antibody responses, while the full biological function should be maintained, quantified by a high binding affinity.

However, extensive sequence modifications within the framework regions (FR) during the trial and error based humanization process often result in reduced or even lost binding affinities (Presta *et al.*, 1993; Adams *et al.*, 2006). This effect may be attributed to critical framework positions within the antibody framework sequence, which stabilize the overall protein structure or the V_H_/V_L_ interface (Chothia *et al.*, 1989), contact the antigen directly (Mian *et al.*, 1991) or establish the Vernier zone (Foote and Winter, 1992) by providing a suitable physico‐chemical environment for a proper conformational ensemble of the CDR loops. In the first step of humanization, the non‐human FR are replaced by carefully selected, appropriate human framework sequences. Afterwards, several critical positions within the human framework have to be backmutated to the non‐human wild‐type. Currently, no universally applicable humanization protocol is available that allows the straightforward, concurrent maintenance of the binding affinity and reduction of the risk for immunogenic responses, i.e. the lowest number of backmutations (BM) necessary.

These choices often have to be made based on empirical knowledge gained from iterative rounds of antibody design, expression and *in‐vitro* binding evaluation on a case‐by‐case basis, making antibody humanization an unpredictable, time‐consuming and costly undertaking. It would hence be highly advantageous to predict the effect of potential BM on the binding affinity of mutants, not only because the mere number of potential candidates is tremendous but also because there is an urgent need to understand the underlying physico‐chemical mechanisms. Yet, the assessment of binding affinities (i.e. the free energy of binding) by computational tools remains a very demanding task. Docking lacks accuracy (mainly because of the imposed rigidity of bigger molecules), while free energy calculations using molecular dynamics (MD) simulations require structural data on the complex and are far from being readily applied to interactions involving a large molecular interface. Nevertheless, *in‐silico* techniques may prove to be a useful addition to the humanization process. In this study, we perform MD simulations to analyse and predict CDR conformations in the humanization process of a mAb. By providing *in‐silico* knowledge from MD simulations, proper decisions about critical BM can be made, before testing the designed variants on the bench. In such a prospective design cycle, many different humanized variants, containing different BM, might be assessed *in‐silico*. The dynamic behaviour of the CDRs is monitored by simulation and expressed in a score that represents the similarity to the wild‐type, the known binder. The most promising mutants are then selected for expression and measurement of binding affinities by experiments. With our technique, we allow for pre‐selection of various humanized variants, thus reducing the amount of required experiments during humanization significantly.

In this study the anti‐idiotypic antibody Ab2/3H6 directed against the broadly neutralizing anti‐HIV‐1 antibody 2 F5 (Muster *et al.*, 1993; Kunert *et al.*, 1998) was used as model protein. It was developed from mouse hybridoma (Kunert *et al.*, 2002) and subsequently chimerized (Gach *et al.*, 2007) or humanized by CDR‐grafting, resurfacing or superhumanization (Mader and Kunert, 2010). Although not eliciting HIV‐1 neutralizing antibodies in first prime/boost studies in BALB/c mice (Gach *et al.*, 2008) or rabbits (Kunert and Mader, 2011), it served as a template for different humanization approaches and MD simulations (de Ruiter *et al.*, 2011) based on the resolved crystal structure (Bryson *et al.*, 2008). The superhumanized variant, su3H6, has lost the binding affinity completely and is therefore suited to be the negative control for the simulation (Mader and Kunert, 2010). An antibody panel consisting of several humanized 3H6 mutants was tested for binding *in‐vitro* to refine a similarity score, quantifying the similarity to the original wildtype antibody (wt3H6). The optimized *in‐silico* system was then tested to predict the influence of BM on the binding affinity in superhumanized variants.

## Methods

### Expression of mAbs

Cell cultures were cultivated in vented 125‐ml Erlenmeyer flasks (Corning) on a climo‐shaker ISF1‐XC (Kuhner) at 140 rpm, 37 °C, 7% CO_2_ and 85% humidity. mAb variants used for training of the MD system (TR01‐TR06) were expressed using stable transfected cell pools of a serum‐free adapted host cell line CHO‐K1 (ATCC CCL‐61) cultivated in ProCHO5 medium (Lonza, Cat. No. BE12‐766Q) supplemented with 4 mM L‐glutamine (Biochrom, Cat. No. K0302), 15 mg/l phenol red (Sigma, Cat. No. P0290) and 0.5 mg/ml G418 (Biochrom, Cat. No. A2912).

To study the effect of BM in heavy chain FR of the superhumanized Ab2/3H6 variants, transient expression was performed in HEK293‐6E cells (NRC biotechnology Research Institute) (Durocher *et al.*, 2002) cultured in F17 medium (Invitrogen, Life Technologies, Cat. No. A13835‐01) supplemented with 4 mM L‐glutamine (Biochrom, Cat. No. K0302), 0.1% Kolliphor P188 (Sigma‐Aldrich, Cat. No. K4894), 15 mg/l phenol red (Sigma‐Aldrich, Cat. No. P0290) and 25 µg/ml G418 (Biochrom, Cat. No. A2912). Transfection of pCEP4 vector (Invitrogen, Cat. No. V044‐50) was mediated by polyethylenimine (PEI) transfection using linear 25‐kDa PEI (Polysciences, Cat. No. 23966).

Culture supernatants were subjected to concentration by either using Amicon Ultra Centrifugal Filters (0.5 ml, NMWCO 10 kDa, Millipore, Cat. No. UFC501096) or Millipore‐Labscale TFF system (Millipore), equipped with a 30‐kDa Pellicon cassette (Millipore, Cat. No. PXB030A50) followed by antibody purification by protein A affinity chromatography using the ÄKTA Purifier Station (GE Healthcare) equipped with a HiTrap MabSelect SuRe protein A column (GE Healthcare, Cat. No. 29‐0491‐04).

### Preparation of mAb variants

The mAb variants used for training of the MD system were expressed in CHO‐K1 cells with stable cell pools and purified by protein A chromatography (TR01 – TR06).

For studying BM in FR of the heavy chain, transient expression was performed in HEK293‐6E cells for different superhumanized Ab2/3H6 variants to allow assessment of re‐established binding affinities. After a 7‐day production phase, crude culture supernatants were concentrated and diluted in ForteBio kinetics buffer.

### Affinity evaluation of mAb variants

All binding studies based on bio‐layer interferometry were performed on a ForteBio Octet QK system (Pall ForteBio) equipped with streptavidin (Pall ForteBio, Cat. No. 18‐5019) or protein A biosensors (Pall ForteBio, Cat. No. 18‐5010).

For immobilization to streptavidin biosensors, purified antibody 2 F5 was biotinylated using the EZ‐Link NHS‐PEG4‐Biotin kit (Thermo Scientific, Cat. No. 21329). Samples and biosensors were equilibrated in kinetics buffer (ForteBio). The streptavidin biosensors were loaded with 20 µg/ml biotinylated 2 F5 antibody, before *k*_on_ and *k*_off_ were measured of purified mAb variants by monitoring association and dissociation in kinetics buffer.

**Figure 1:**
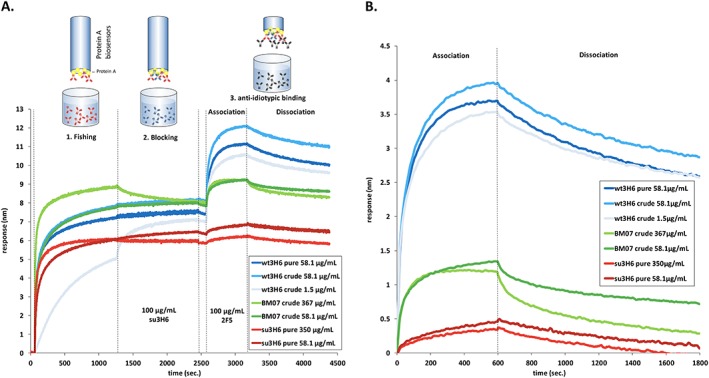
Protein A fishing from crude culture supernatants by bio‐layer interferometry. The ForteBio Octet system was equipped with protein A biosensors to immobilize transiently expressed humanized Ab2/3H6 mutants from concentrated and crude culture supernatants. (A) Real‐time sensorgram of wt3H6, su3H6 and BM07 at different concentrations. Assay‐step times were as follows: 60‐s baseline in kinetics buffer, 1. Fishing: 1200‐s immobilization of antibodies from crude culture supernatants, 2. Blocking with 100 µg/ml purified mAb su3H6 for 1200 s, followed by 120‐s baseline/washing in kinetics buffer, 3. Binding measurements: 600‐s mAb 2 F5 association (100 µg/ml) with immobilized Ab2/3H6 variants, followed by 1200‐s dissociation in kinetics buffer only. (B) Association and dissociation curves extracted from raw data and aligned to baseline by the fortebio software.

Capturing mAbs from crude culture supernatants for quantification already has been proven as an established and robust procedure (Tobias and Kumaraswamy, 2014). In this work we further demonstrate the use of protein A biosensors to determine the binding affinity of the immobilized antibodies to its antigen (anti‐idiotypic antibody). Protein A sensors were equilibrated in kinetics buffer for 60 s before transiently expressed Ab2/3H6 variants were immobilized from the crude and concentrated culture supernatants for 1200 s (Figure 1A). This time period for capturing antibodies from crude culture supernatants was considered as a good trade‐off between reaching a suitable sensor saturation level and overall assay time. To block possible free protein A binding sites a blocking step of 1200 s was introduced by submersing the loaded protein A biosensors in high concentration (100 µg/ml) of purified mAb su3H6, showing no interaction with 2 F5. Following a baseline/washing step for 120 s, the association of purified target antibody mAb 2 F5 (100 µg/ml) to protein A‐immobilized Ab2/3H6 variants was measured followed by a dissociation step in kinetics buffer only. mAb 2 F5 binding to anti‐idiotypic mutants showed a high response for wt3H6 samples of about 3.5 nm in the baseline corrected sensorgram (Figure 1B). Both crude and pure wt3H6 preparations at high concentrations gave similar binding curves. Additionally, also with a very low initial wt3H6 concentration (1.5 µg/ml) a high binding response could be observed reaching a response level of 3.4 nm. These results demonstrate that it is possible to distinguish mAb 2 F5‐binders (i.e. wt3H6) from non‐binders (i.e. su3H6), the latter of which gave only a very low non‐specific response level probably resulting from minimal residual free protein A binding sites. From the experimentally obtained *K*_d_ values, the average binding free energies were calculated to be compared to the score obtained from the simulations. To eliminate outliers, data sets have been excluded for which the fit to the theoretical signal curve was calculated to have a correlation coefficient (*R* ^2^) below or equal to 0.8. Afterwards, binding free energies were calculated using Δ*G*_binding_ = *k_B_T* ln(*K*_d_) for all remaining measurements. Measurements, deviating more than 5.6 kJ/mol from the average (factor 10 in *K*_d_) were iteratively excluded from the calculation. From the remaining values (at least three measurements for each variant), the average binding free energy is reported.

### *In‐silico* score calculation

**Figure 2:**
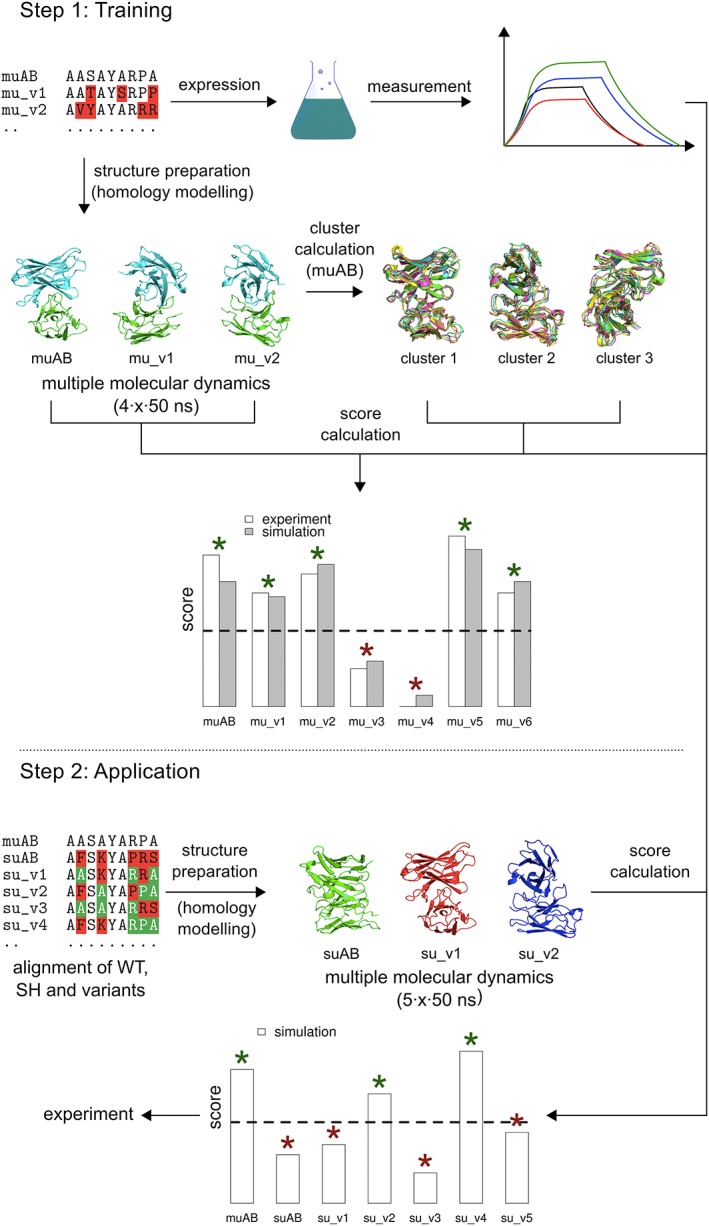
Workflow of the simulation assisted humanization approach. In the training step (above), molecular dynamics simulations of the murine derived wild‐type and selected mutant variants are performed and assessed in terms of a score (see equation 1), representing similarity to the wild‐type loop conformations. The same set is expressed, and affinities are measured experimentally, allowing for the identification of the qualitative boundary separating binders from non‐binders (the latter marked with a red asterisk). The second step (below) starts with the superhumanized antibody variant, and a set with selected backmutations. With the same procedure as before, a computational score can be calculated holding qualitative information on the expected binding affinities. The cutoff determined in the previous step can now be used to classify the results. Note, that this is only an illustrative diagram; the actual values obtained throughout this study are reported in Figures 5 and 6 and Table 1.

The approach presented here relies solely on the structural and dynamic information retrieved from the monoclonal antibody, as shown in Figure 2. From multiple simulations on the murine antibody, we obtain the most prominent conformations of the CDRs, represented by the central member structures (CMS) of conformational clusters. Subsequently, variants are simulated, and the reproduction of the wild‐type reference conformations (CMS) is expressed through a similarity score. It is based on time series of the root‐mean‐square deviation (RMSD) of the CDR atoms (fitted to the flanking framework backbone; see below) with respect to the wild‐type CMS. This means that the score is higher for variants, which are closer to the original rodent antibody in terms of their structural ensembles. The score is calculated according to,

(1) $$\text{score}=\frac{100}{s\cdot m\cdot a}\sum_{i=1}^{a}\sum_{j=1}^{s}\sum_{k=1}^{n}\sum_{x=1}^{m}\left\{\begin{array}{ll} 1, & {\text{RMSD}}_{x,{CMS}_{i}}\leq c_{k}\\ 0, & \text{else} \end{array}\right.$$

where *c* is a vector of thresholds used, *RMSD_x,CMS_* is calculated for a given configuration–CMS pair (in nanometers), *m* is the number of configurations in a trajectory, *n* is the number of thresholds considered, *a* is the number of significant clusters and *s* is the number of replicate simulations for this very variant. In an initial training round, the scores are compared to the binding free energy, experimentally determined by affinity measurements for some variants, to estimate a cutoff of the similarity score. In the second stage, BM of the superhumanized variant are simulated until a candidate with a score above the cutoff is identified, which can then be further optimized. Our approach is based on the assumption that mutants with comparable structures and dynamics as the original monoclonal antibody also show significant binding. Obviously, the reverse statement is not necessarily true as other conformations/ensembles may bind as well or even better but are disregarded in our approach because they were not present in the murine reference. Furthermore, we assume that induced fit effects upon binding play a minor role and the relevant pre‐binding conformations will be sufficiently sampled in the MD simulations, following the conformational selection paradigm (Lee and Craik, 2009; Vogt and Di Cera, 2013).

**Table 1:** *In‐silico* score predictions for all simulated variants based on the (non‐binding) su3H6 antibody

Variant	Score
su3H6	1.0
BM01	2.6
BM02	3.3
BM03	3.5
BM04	1.7
BM05	1.8
BM06	2.8
BM07	2.8
BM08	1.3
BM09	2.9
BM10	0.8
BM11	3.2

**Figure 3:**
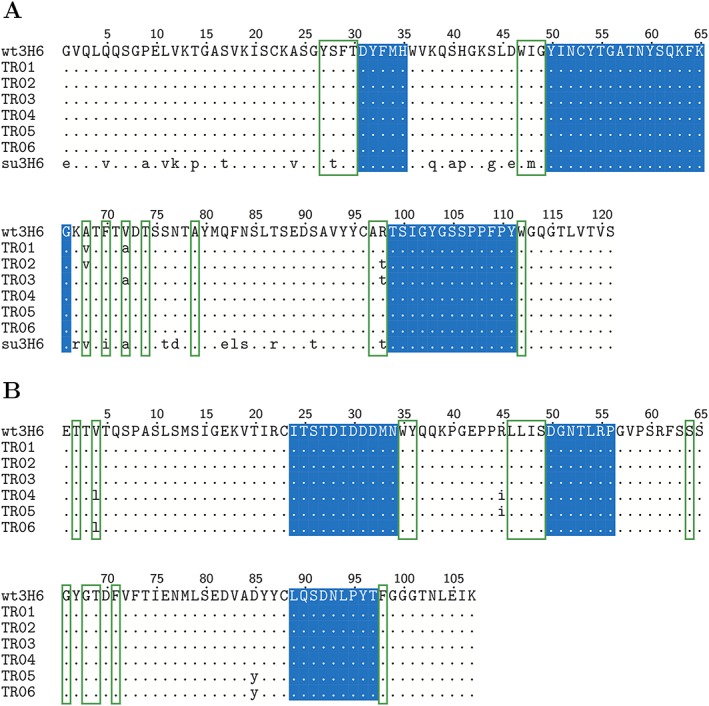
Sequence alignment of the heavy (A) and light (B) chain of the wild‐type (wt3H6), the training (TR01–TR06) and the superhumanized (su3H6) variant. The CDR regions as defined by Kabat were conserved during the humanization process and kept constant in all variants throughout this study (blue background). Identical amino acids compared to the murine/wild‐type template sequence are indicated as dots. Vernier zone residues of the heavy chain defined by Foote and Winter are marked by green frames.

**Figure 4:**
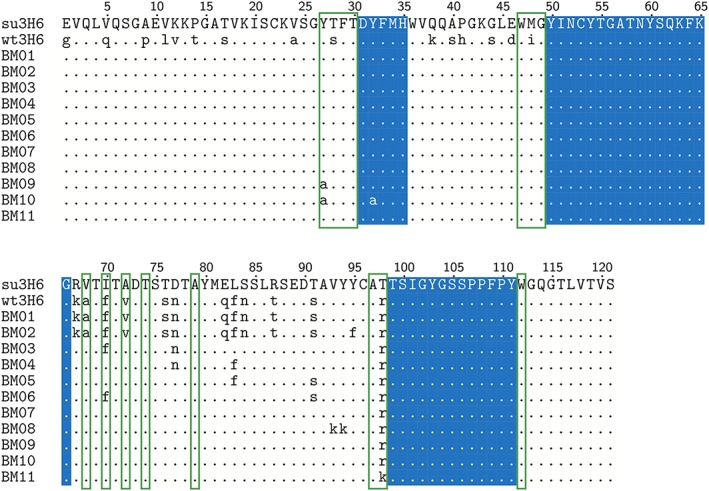
Sequence alignment of the heavy chain of the wild‐type (wt3H6), the superhumanized (su3H6) and the simulated (predictive, BM01–BM11) variants. The CDR regions as defined by Kabat were conserved during the humanization process and kept constant in all variants (but BM10) throughout this study (blue background). Identical amino acids compared to the murine template sequence are indicated as dots. Vernier zone residues of the heavy chain defined by Foote and Winter are marked by green frames. No light chain mutations in respect to the superhumanized variant were applied to the backmutation variants.

We have applied this workflow to the murine/wild‐type anti‐idiotypic Ab2/3H6 antibody, which is directed against the broadly neutralizing anti‐HIV‐1 antibody 2 F5 and has been studied by our groups earlier (Kunert *et al.*, 1998; Kunert *et al.*, 2002; Mader and Kunert, 2010; de Ruiter *et al.*, 2011). The binding to mAb 2 F5 is mainly facilitated by the third CDR loop of the heavy chain (Ab2/3H6), which simplifies the analysis. In order to cover cases that require multiple loops for proper binding, the above equation can readily be extended. We defined six training variants based on critical framework positions (Supplementary Table S1) of the murine/wild‐type Ab2/3H6 (TR01 to TR06; Figure 3), to train the scoring function and 11 backmutation variants based on the non‐binding su3H6 antibody (BM01 to BM11; Figure 4), with mutation sites in the FRs selected based on their location in the X‐ray structure, vicinity to the Vernier zone, or being in the V_H_/V_L_ interface region.

### MD simulations

For wt3H6 (the binding reference), six simulations (of 100 ns each) of the V_H_/V_L_ complex were undertaken. For the compounds in the training set, four simulations (replicates) for each variant were performed with a trajectory length of 50 ns, respectively. The superhumanized version and its derivatives have been simulated five times each (50 ns). Initial coordinates were taken from the crystal structure of the 3H6–2F5 complex (PDB ID: 3BQU) (Bryson *et al.*, 2008); variants were modelled using the programme MOE (MOE, 2014). The simulations were performed without any restraints. All simulations were performed using the GROMOS (Christen *et al.*, 2005; Schmid *et al.*, 2012) simulation package with the 54A7 parameter set (Oostenbrink *et al.*, 2004; Schmid *et al.*, 2011) in a sufficiently large water‐box (0.8‐nm minimum solute to box–wall distance). Counter‐ions were added (Na^+^ and Cl^−^) to neutralize the net charge in the box, up to a limit of 15 ions of each type, e.g. to a solute with net charge −9 *e*, 15 Na^+^ and 6 Cl^−^ were added. The rectangular periodic simulation boxes (roughly 5.5 × 6.5 × 7.5 nm) contained approximately 27 000 atoms. Prior to the production runs, the systems were equilibrated from 60 K to 300 K in six discrete steps with a simulation length of 20 ps each and a weak thermostat‐coupling with two baths for the solute and solvent (relaxation time of 0.1 ps) and weak barostat‐coupling (relaxation time of 0.5 ps and an isothermal compressibility of 4.575 × 10^−4^ (kJ mol^−1^ nm^−3^)^−1^). In order to avoid artifacts originating from the same starting structure, only the last 40 ns of each trajectory has been analysed, while for cluster analysis the last 90 ns of the wt3H6 replicates has been used. All simulations in the training set were extended to 100 ns (90 ns analysed) without a significant change in the subsequent analyses (Supplementary Figure S1); therefore, an additional replicate was preferred over longer simulation times in the prediction set. For consistency with the superhumanized and predicted variants, all the reported data is based on the last 40 ns of the first 50 ns of the training set simulations. Weak temperature and pressure coupling (Berendsen *et al.*, 1984) ensured a constant temperature of 300 K and a constant pressure of 1 atm, respectively. SHAKE (Ryckaert *et al.*, 1977) was used to maintain the bond distances at the energy minimum. The integration time step used was 2 fs. Interactions within 0.8 nm were calculated at every time step from a pairlist that was updated every five steps. Intermediate range interactions up to a distance of 1.4 nm were calculated at pairlist updates and kept constant between updates. Long range interactions were approximated with a reaction field contribution (Tironi *et al.*, 1995) to the energies and forces, accounting for a homogeneous medium with a relative dielectric constant (Heinz *et al.*, 2001) of 61 beyond the cut‐off of 1.4 nm.

### Fitting procedure

In order to compare the conformational ensembles generated by the MD simulations to one another, the respective backbone atoms of FR 3 and 4 of the heavy chain (H:FR3 and H:FR4) were used for a roto‐translational least‐squares fit. The RMSD calculation afterwards (both for the clustering and the score calculation) was based on all atoms (including side‐chain atoms) of complementary determining region 3 of the heavy chain (H:CDR3).

## Results and Discussion

**Figure 5:**
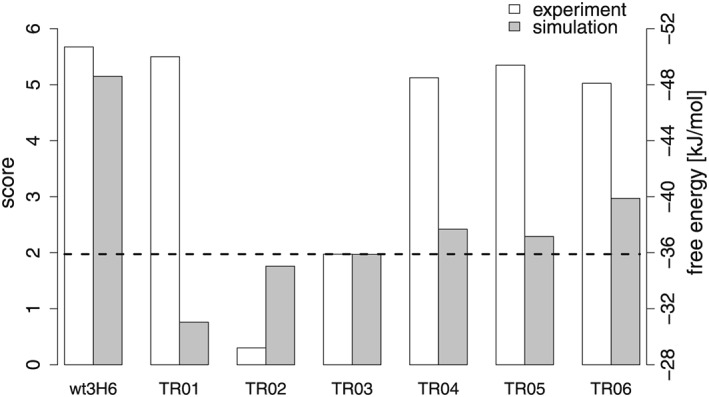
Similarity scores and free energies of binding for the variants. For the calculation of the score, see equation 1. The grey bars indicate a score for the similarity to the murine/wild‐type 3H6 antibody (left axis), while the white bars indicate experimentally determined binding free energies (right axis). For TR01, the simulation score and the experimentally determined binding free energy are clearly disagreeing, see main text.

All MD simulations lead to stable trajectories with backbone atom‐positional RMSD values to the initial structure of, on average, 0.3 (±0.1) nm for the training set and 0.3 (±0.1) nm for the superhumanized variants (Supplementary Figures S2 and S3). The secondary structure elements of the FR (anti‐parallel beta sheets) were maintained throughout. Averaged over simulation time and the FR residues, which are also in β‐sheet conformation in the crystal structure, the occurrence of β‐sheet conformations amounted to 88% (±2%) for the training set and 83% (±4%) for the superhumanized variants as determined by the determine secondary structure of proteins (DSSP) algorithm (Supplementary Figure S4). To calculate the reference structure(s), that are most representative of the conformational ensemble of H:CDR3 in wt3H6, we calculated the cross RMSD matrix of structures collected from all its replicates. A clustering algorithm was applied, using a cut‐off of 0.2 nm (Daura *et al.*, 1999). The majority of configurations belonged to the first cluster (52%), while the others were only populated by small amounts (up to 15%), suggesting to use a single representative structure, i.e. a = 1 in equation 1. Without loss of generality, the subsequent analysis could have included additional clusters. Subsequently, the similarity score was computed for the training set, which is shown in Figure 5 together with the experimentally determined binding free energies. From the MD simulations, TR01, TR02 and TR03 were most dissimilar to the original wt3H6. Indeed, for TR02 and TR03 the binding affinity seems to be reduced by 15 – 20 kJ/mol, while the mutations applied to TR04 to TR06 do not seem to affect affinity. Only for TR01, there is no match between the similarity score and the measured binding affinity to 2 F5, possibly because of alternative binding modes (see above).

**Figure 6:**
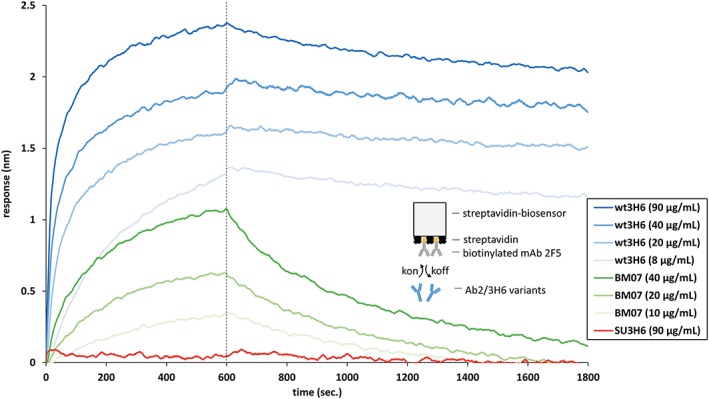
Real‐time bio‐layer interferometry sensorgrams for determination of anti‐idiotypic binding affinities of purified Ab2/3H6 variants to its target antibody mAb 2 F5. Streptavidin biosensors were loaded with biotinylated mAb 2 F5 (20 µg/ml) followed by a washing/baseline step. Association (600 s) and dissociation (1200 s) of different Ab2/3H6 variants were measured at different concentrations or buffer only, respectively.

A superhumanized variant (su3H6) was described earlier, which lost binding affinity completely (Figure 6) (Mader and Kunert, 2010). Our MD simulations confirm that the CDR is significantly different (similarity score of 1.0). Eleven candidates with specific BM (BM01 to BM11) were proposed and are represented in Figure 4. The associated similarity scores for these variants are given in Table 1. Based on the results of the training simulations, the emphasis for the BM was placed on the FR 3 of the heavy chain. In the first variant, BM01, the entire FR was backmutated to the murine/wild‐type sequence. The similarity score was significantly lower than for wt3H6, indicating that BM01 is not a variant that is to be tested experimentally. Interestingly, variant BM02, which contains the additional mutation Y95F, leads to a significantly higher similarity score. The side‐chain of Y95 is pointing into the interface region between the V_L_ and V_H_ domains, suggesting that the packing of amino acids at the interface and in the hydrophobic core plays a crucial role. To maintain optimal packing with the human residues from the other FR, variants with fewer BM were proposed in the form of BM03 to BM07. The most interesting variant in this respect is variant BM07, in which only a single backmutation, T98R, was applied and for which a reasonably high similarity score was observed. Next, we proposed an artificial double mutation to lysine at positions 93 and 94 in variant BM08 to have a non‐binding variant (negative control) that still contains the T98R mutation. Indeed, the similarity score dropped considerably.

Variant BM07 was transiently expressed using the HEK293–6E system. Because of low transient expression titers the affinity to 2 F5 was measured by a modified protein A fishing setup (see above): Protein A biosensors were immersed in the supernatant to bind the transiently expressed variant. After blocking the remaining binding sites of the sensors with inactive su3H6, the antigen 2 F5 was added, and binding and dissociation could be measured. This superhumanized variant, containing only a single backmutation (BM07), showed a significant improvement in anti‐idiotypic binding affinity to mAb 2 F5 resulting in final response levels of 1.4 nm at two different concentrations. Although the binding affinity of BM07 did not reach the full binding capabilities of wt3H6, it showed a significant increase with respect to its precursor molecule, i.e. the non‐binding su3H6 antibody (Figure 1B). Based on these qualitative results we expressed BM07 on a larger scale followed by protein A chromatography purification and quantitative assessment of its binding properties using the streptavidin bio‐assay setup (Figure 6). The results of the two different methods were qualitatively comparable (Δ*G*_binding_ = −43.5 kJ/mol for the first and Δ*G*_binding_ = −38.7 kJ/mol for the latter), indicating that our method for quickly estimating binding affinities from the supernatant is reasonable. Unfortunately, difficulties with expression efficiency have so far prevented the validation of additional superhumanized variants.

**Figure 7:**
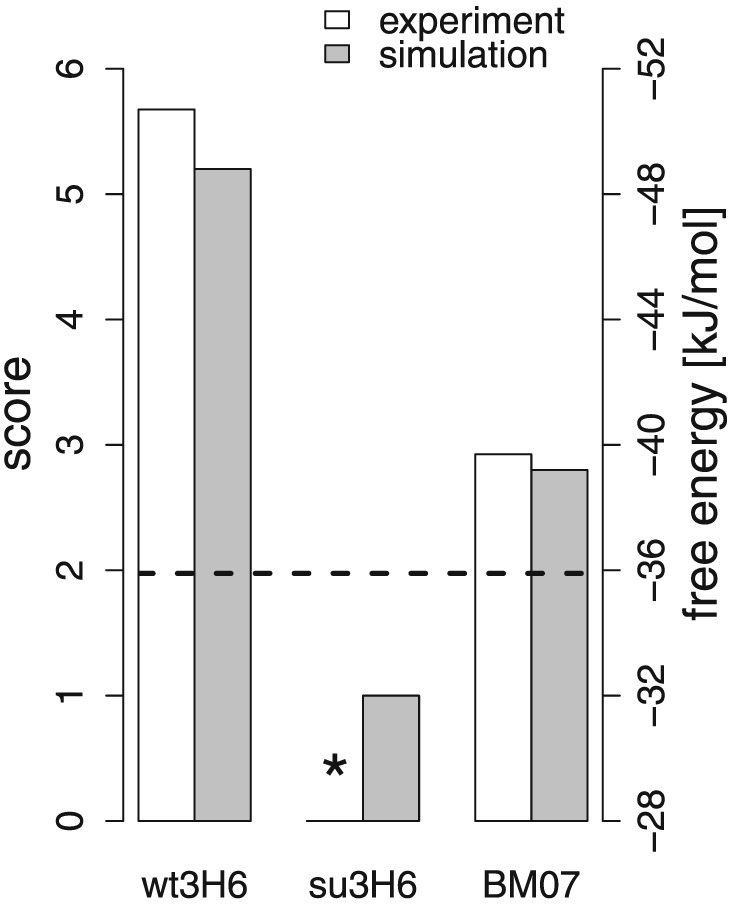
Similarity scores and free energies of binding for the wild‐type, the superhumanized antibody and BM07. The experimental binding free energy of su3H6 was below the detection limit.

**Figure 8:**
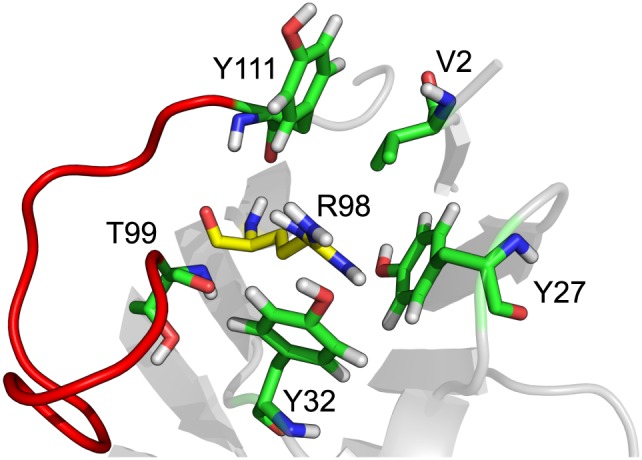
The importance of R98 is likely to be explained by its interaction with the surrounding tyrosines through cation–pi interactions, most notably with one located in CDR loop three (Y111). Therefore, it is of utmost importance that this position remains an arginine in order to retain binding affinity. The “tyrosine cage” at that position could, in conjunction with R98, lead to a more restricted local environment for the CDR loop, which is shown in red.

Both our simulations and the experiments show, that the backmutation T98R is sufficient to restore binding affinity to a significant extent (Figure 7). This confirms that a single backmutation can be sufficient to (partially) restore binding affinity for superhumanized variants and also indicates a crucial role for R98, which could be successfully predicted by our *in‐silico* approach. In fact, 80% of the human heavy chain germline gene sequences retrieved from the IMGT/Gene‐DB databank (Lefranc *et al.*, 2005) have an Arg at this very position (Supplementary Table S2). By replacing this residue by threonine, as in su3H6, TR02 and TR03, binding affinity is severely reduced. From the simulations we observe that the charged side chain of R98 interacts with the Y27, Y32, Y111 side‐chains (in some sort of “tyrosine‐cage”) and the T99 backbone (Figure 8). It seems, that through the cation–π interactions in this structural region, certain conformational restrictions are applied to the CDR3 loop (heavy chain), which are crucial for binding. To test this hypothesis, we also investigated other variants computationally (BM09 to BM11; Table 1, Figure 4) which will be the subject of future experimental validation studies. It is remarkable that the double mutation of Y27 and Y32 (BM10) to alanine is predicted to lead to a severe loss of binding, while the single mutation of Y27 (BM09) seems to make no difference. Moreover, a substitution of R98 by lysine (BM11), which also contains a positively charged moiety at a comparable distance to the backbone, seems to be able to function appropriately.

## Conclusion

We conclude that the presented approach, calibrated with experimental data, allows for useful predictions of the effect that distinct BM have on the binding affinities of antibody variants. We have validated our predictions using well‐established experimental techniques and also shown the qualitative agreement of these results with a newly developed efficient method, based on cell culture supernatant. Our computational workflow can be applied as an ab‐initio protocol; however, additional information, such as the relative importance of the respective CDR loops in binding, might be included. Moreover, simulations allow for a molecular rationalization of the observed differences, which may help to guide further rounds of compound design where necessary. The approach is readily applicable to different antibodies as only structural information on the original antibody is required and no explicit binding free energy calculations are performed. Presumably, iterative cycles of improvement will be necessary to re‐establish a binding affinity, comparable to that of the wild‐type, where promising combinations of the previous round are natural candidates.

## Supporting information

Supporting info item
