# Supplementary material for: Antibody humanization by molecular dynamics simulations—in‐silico guided selection of critical backmutations
Source: J Mol Recognit. 2016 Jan 8;29(6):266–75. doi: 10.1002/jmr.2527 (PMC4948679; doi:10.1002/jmr.2527)
Supplement: Supplementary file 1 — Supporting info item [file JMR-29-266-s001.pdf]

Supplementary material: Antibody humanization by molecular dynamics simulations – in-silico guided selection of critical backmutations

Christian Margreitter,[#a] Patrick Mayrhofer[#b], Renate Kunert[b] and Chris Oostenbrink*[a]

Content:

Table S1: properties of selected amino acid side-chains, mutated in the training set.

Table S2: Occurrences of amino acids at the position equivalent to R98 in human germline genes.

Figure S1: Comparison of computational scores from 40 ns and 90 ns trajectories for the training set.

Figure S2: Average atom-positional root-mean-square deviations over the last 20 ns of all replicates for the training set.

Figure S3: Average atom-positional root-mean-square deviations over the last 20 ns of all replicates for the superhumanized variants.

Figure S4: β-strand fractions over the last 40 ns of all replicates for the training set.

a C. Margreitter, Dr. C. Oostenbrink
Institute of Molecular Modeling and Simulation
University of Natural Resources and Life Sciences
Muthgasse 18, 1190 Vienna
E-mail: chris.oostenbrink@boku.ac.at

b P. Mayrhofer, Dr. R. Kunert
Department of Biotechnology
University of Natural Resources and Life Sciences
Muthgasse 18, 1190 Vienna
E-mail: renate.kunert@boku.ac.at

# Both authors contributed equally to the work.

* To whom correspondence should be addressed.

wt3H6	properties	su3H6	properties	possible function	position in crystal
A68^{HC}	small, aliphatic, hydrophobic	V68^{HC}	aliphatic, hydrophobic	backmutated in GC3H6, part of Vernier zone	surface, facing towards CDR-H2/beta-sheet (stabilizing barrel?)
V72^{HC}	aliphatic, hydrophobic	A72^{HC}	small, aliphatic, hydrophobic	backmutated in GC3H6 and GA3H6, defining canonical CDR structure class, part of Venier zone	surface, underneath CDR-H2
R98^{HC}	basic	T98^{HC}	polar, uncharged	backmutated in GC3H6 and GA3H6, defining canonical CDR structure class, part of Vernier zone	between CDR-H1 and CDR-H3 (stabilizing Ag binding site)
V4^{LC}	aliphatic, hydrophobic	L4^{LC}	aliphatic, hydrophobic	backmutated in GC3H6, part of Vernier zone	surface, facing towards CDR-L3/beta-sheet (stabilizing barrel?)
R45^{LC}	basic	I45^{LC}	aliphatic, hydrophobic	backmutated in GC3H6, buried in V_H/V_L interface, not part of Vernier zone	surface, interaction with CDR-L2?
D85^{LC}	acidic	Y85^{LC}	aromatic, hydrophobic	backmutated in GC3H6 and GA3H6, affects overall protein stability	interaction with Kappa constant region?

Table S1: Properties of amino acid side-chains selected for establishing wt3H6 double mutants used as a training panel (TR01-06) for MD simulations. The critical functions of these framework positions are described in Mader and Kunert, Protein Eng. Des. Sel. PEDS, 23, 947 – 954 (2010). Spatial positions in the crystal structure 3BQU are summarized.

**Search parameters:**	Species:	homo sapiens
	Gene type:	variable
	Functionality:	functional
	Locus	IGH
	Molecular component:	IG

Residue	Number of alleles	% of total
Arginine (R)	175	80%
Threonine (T)	10	5%
Lysine (K)	24	11%
Histidine (H)	7	3%
Alanine (A)	2	1%
Total	218	100%

Table S2: Conserved amino acid residues at the position equivalent to R98 in the human IGHV germline genes (IMGT/Gene-DB).

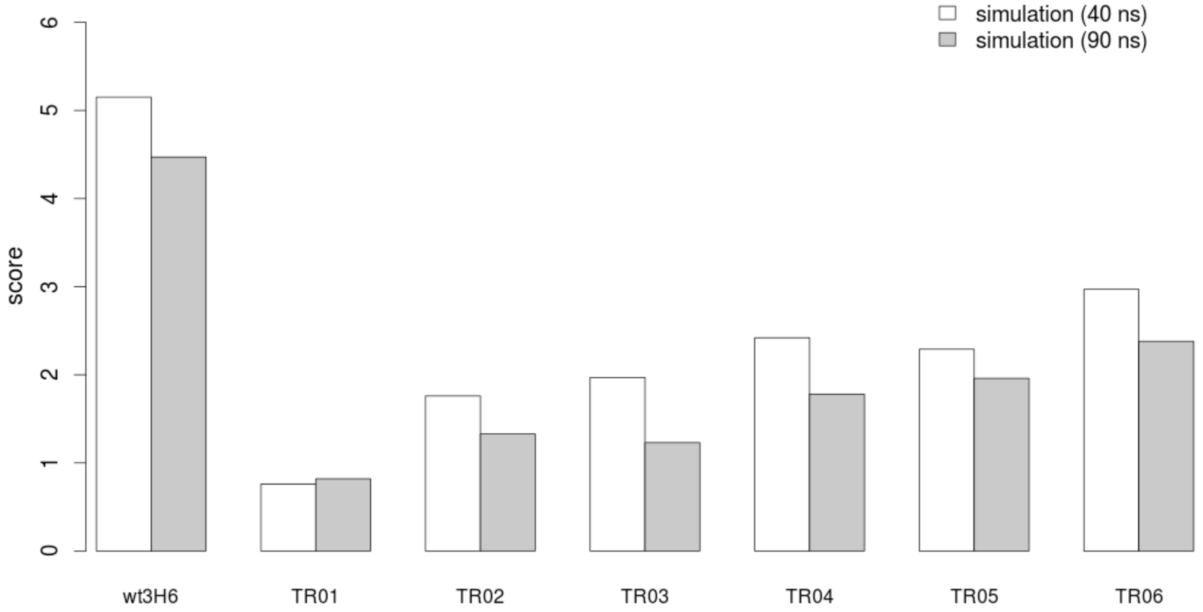

Figure S1: Use of 90 ns trajectories for the score calculation results in a slightly lower absolute score for the variants, while retaining the relative, significant differences. Therefore, it seems reasonable to favor a higher number of replicates per variant over a individual simulation time larger than 50 ns.

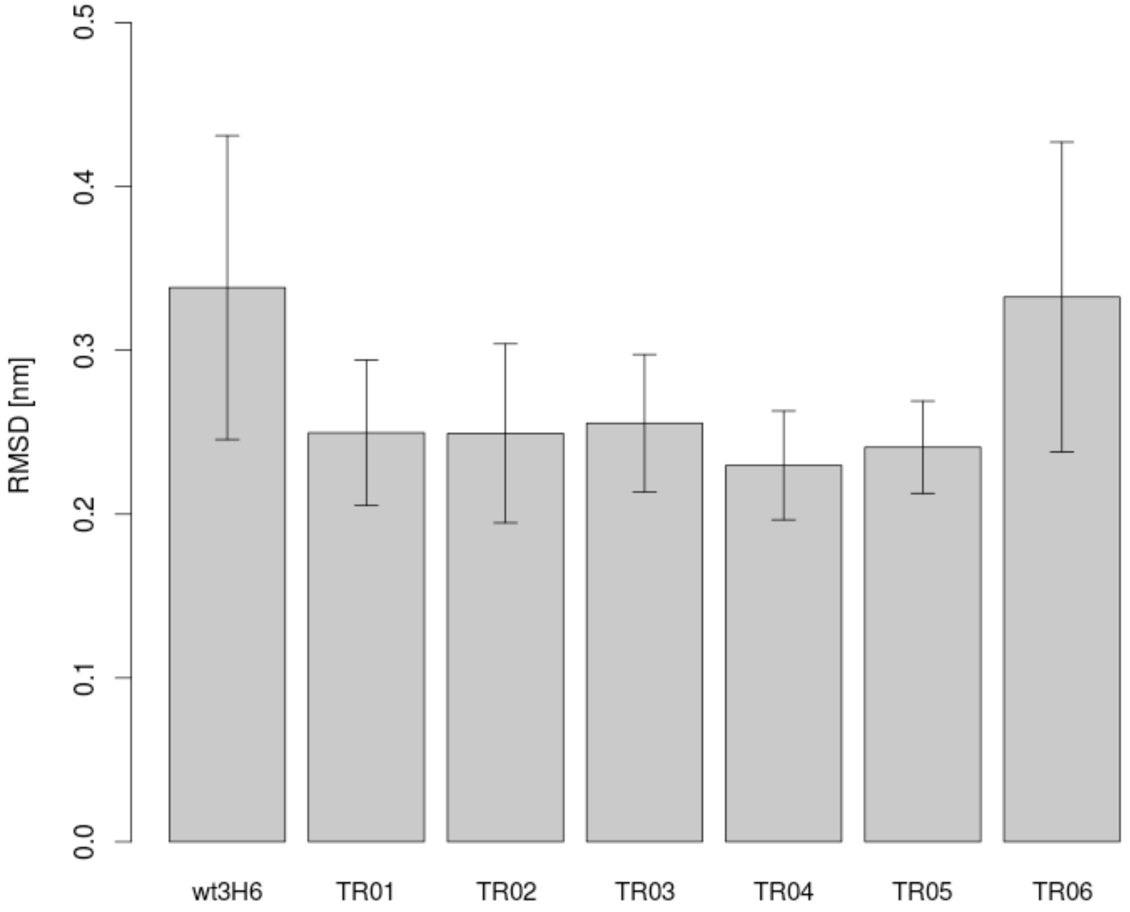

Figure S2: The average backbone atom root-mean-square deviation (RMSD) with respect to the crystal structure over the last 20 ns of all replicates for the training set. The RMSD is calculated for all backbone atoms of the framework regions after a least-squares fit on these atoms. The error bars indicate the standard deviation.

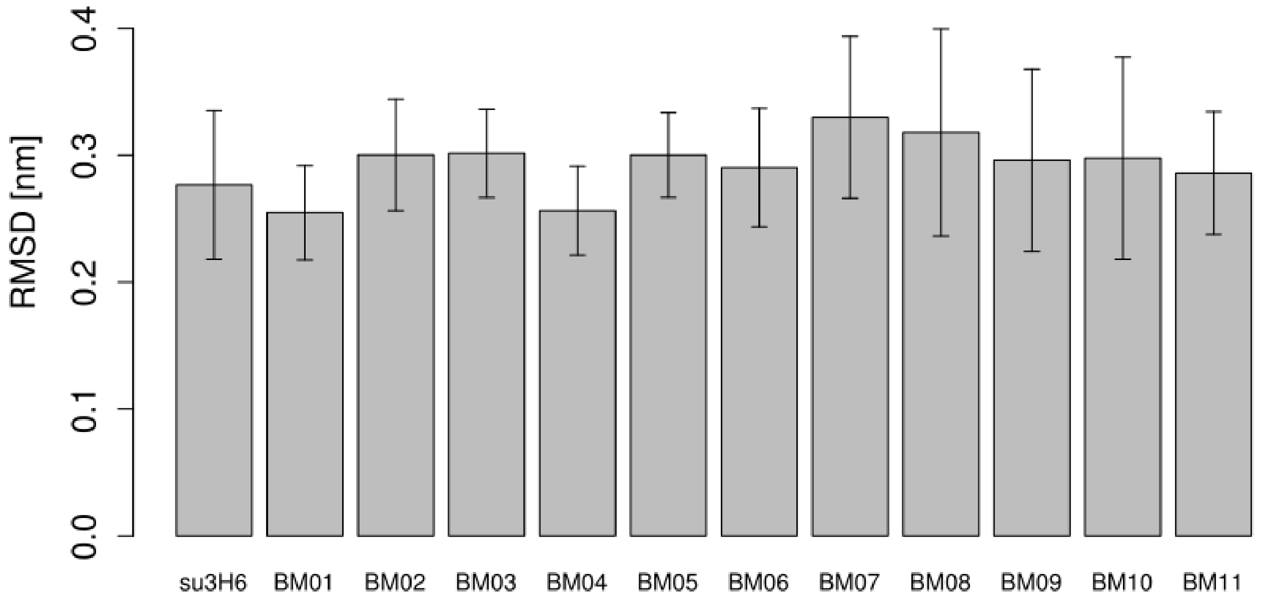

Figure S3: The average backbone atom root-mean-square deviation (RMSD) with respect to the crystal structure over the last 20 ns of all replicates for the superhumanized variants. The RMSD is calculated for all backbone atoms of the framework regions after a least-squares fit on these atoms. The error bars indicate the standard deviation.

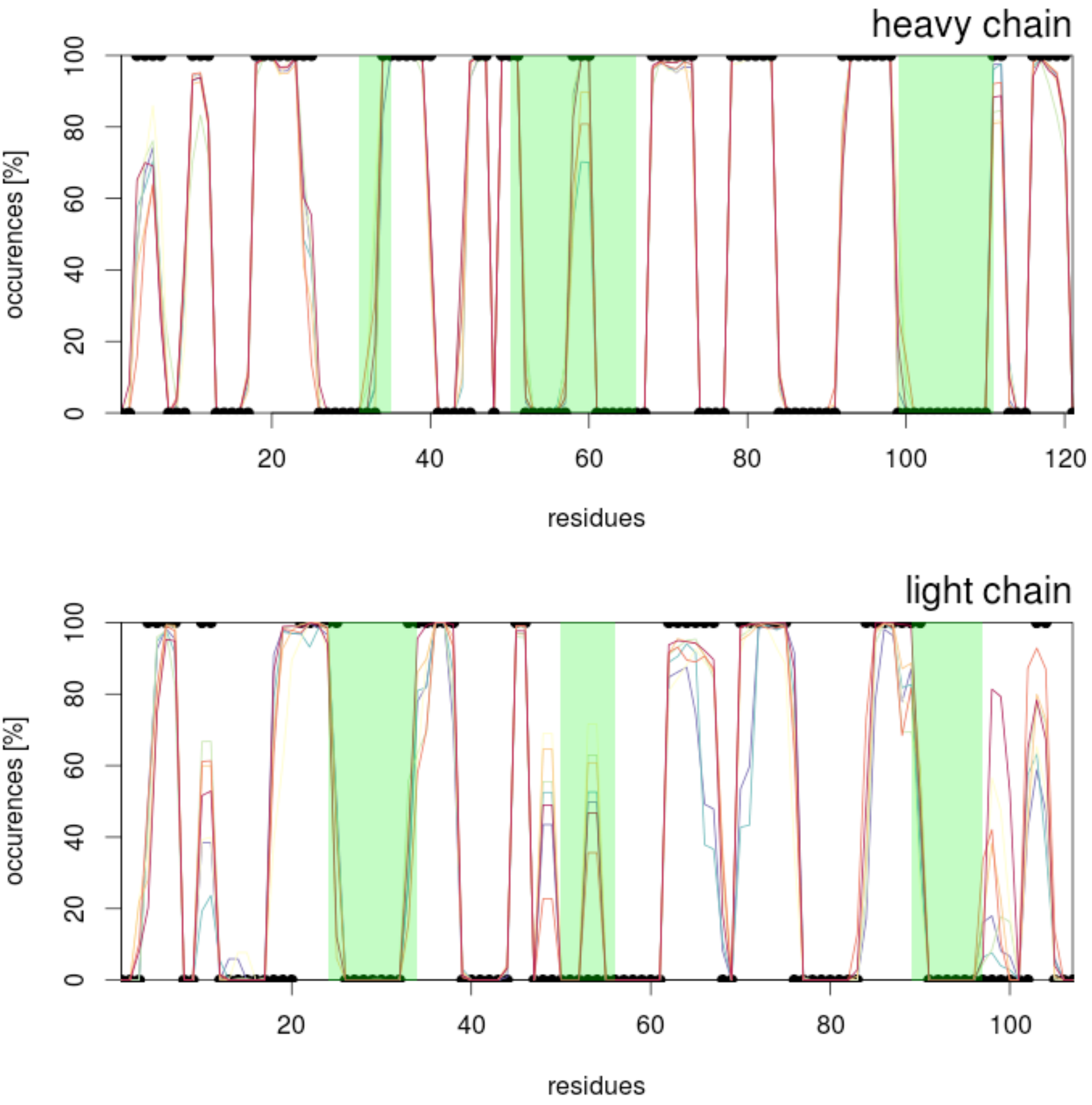

Figure S4: β-strand average occurences of conformations over the last 40 ns over all replicates for the training set as determined by the program DSSP for the framework and complementary-determining regions (CDRs; in green). The classification of the crystal structure is shown in black dots (either 100% or 0%), which agrees well with the mean values of the trajectories. This indicates a stable secondary fold throughout the simulation.
